# Parental Knowledge and Its Impact on Nutrition and Oral Health Habits in Children: A Cognitive Investigation

**DOI:** 10.3390/jcm13185631

**Published:** 2024-09-23

**Authors:** Fabrizio Guerra, Giulia Zumbo, Lucia Straccamore, Laura Sansotta, Claudio Stamegna, Roberta Iacono, Denise Corridore, Iole Vozza

**Affiliations:** Department of Oral and Maxillo-Facial Sciences, “Sapienza” University of Rome, Via Caserta 6, 00161 Rome, Italy; fabrizio.guerra@uniroma1.it (F.G.); straccamorelucia@gmail.com (L.S.); sansotta.2041402@studenti.uniroma1.it (L.S.); stamegna.1839217@studenti.uniroma1.it (C.S.); iacono.roberta@libero.it (R.I.); denise.corridore@uniroma1.it (D.C.); iole.vozza@uniroma1.it (I.V.)

**Keywords:** nutrition, oral health, weaning, awareness, oral health prevention, pediatric dentistry

## Abstract

**Background**: The correlation between the intake of certain nutrients and the development of oral pathologies has been demonstrated by several studies. The objective of this epidemiological investigation was to evaluate parents’ knowledge and awareness of children’s nutrition starting from the early stages of childhood. **Methods**: A questionnaire with 35 questions was handed over to 120 parents of infants aged 4 to 24 months. Among these, 20 questionnaires were excluded because they were incomplete; hence, the final sample analyzed consisted of 100 questionnaires. The outcome of this study revealed that the parents are well aware of many common topics, but their knowledge regarding specific prevention measures, i.e., the use of systemic fluoride as per the international guidelines, needs proper attention. **Results**: and **Conclusions**: After analyzing all the collected data, it is possible to conclude that prevention is the best investment to avoid the onset of the carious pathology, which can be possible through parental education, for example, by raising awareness among expectant mothers, through different actions in the territory. However, further research is needed as this study has some limitations and the convenience sample is hospital-based and not effectively representative of the whole population.

## 1. Introduction

Pediatric age represents a crucial phase for the development of an individual from the very first months of life, and it represents a key stage in which individuals can effectively intervene with adequate health promotion policies since parents could be unprepared for this delicate role [1].

In the first place, an increasingly broad collaboration with pediatric dentists, pediatricians, kindergarten educators, the healthcare staff of hospital pediatric care, and the maternal-infantile services of local health authorities is fundamental to improve parents’ psicopedagogic education. This cooperation allows the establishment of a multidisciplinary network of experts with the aim of improving intervention methodologies for the health of children. Prevention carried out in appropriate centers needs to assume a primary role in reducing the incidence of dental decay and other diseases related to this age group. In addition, it has become increasingly important to reevaluate the educational role of school refectories from the first years to introduce children to an adequate and balanced nutritional regime [2].

It is well known that correct nutrition implies a true, deeply rooted knowledge of the basic concepts of nutritional needs, the proper balance of nutrients, the correct qualitative and quantitative food distribution, and the child’s right body image. The utmost attention needs to be given to the weaning phase to reduce the incidence of allergies, food intolerance, dental anomalies, overweight, and malnutrition in the first years of life, which are often associated with early weaning and inadequate food products for the age of the child [3,4,5,6].

Finally, it is necessary to reiterate that tooth decay is the most common oral disease. However, it is a common belief that primary dentition does not influence permanent dentition. According to this erroneous concept, preventive measures suitable for the maintenance of pediatric patients’ oral health are often not implemented [7]. This argument has been proven with a survey administered to parents whose children were 4 to 24 months old, which consisted of thirty-five questions. Furthermore, nutrition is impacted not only by parental knowledge but also by the socioeconomic level of families and cultural habits, among other factors.

The questionnaire collected information about toddlers’ eating habits during the weaning period, oral hygiene habits before and after the dental eruption, and essential knowledge on the use of systemic and/or topical fluoride in addition to the amount consumed with daily meals.

In addition, the goal of this study was not only to provide parents with valid clarification about nonharmful fluoride values and concentrations that can be found in pediatric toothpastes, but also to provide them with guidelines about when fluoride should be taken regularly by their children [8,9].

The argument at issue stems from the necessity of investigating the relatives’ awareness of the nutrient composition of foods such as meat/fish or fruit homogenized baby food, freeze-dried baby food, rice creams, flours, baby food, etc., and the possible addition of sugar. 

The aim of this work was to collect information about the influence that a medium-level socioeconomic and cultural context can have on parents’ lifestyle, behavior, and nutritional choices toward their children. In particular, this research consists of a synthesis of the main factors responsible for the onset of any pathological conditions concerning the oral cavity. In fact, these behaviors enacted by the parents could contrast with the WHO guidelines, which are advisable to follow. Therefore, these habits implemented by the parents themselves could result in the development of incorrect habits in their children. The survey is based on the principles of involvement and empowerment, which facilitate health choices and result in effective benefits, such as children’s healthy growth since the neonatal period. In particular, the utmost attention has been given to the knowledge of correct nutrition and to the appropriate oral hygiene practices carried out by parents for children aged 4 to 24 months.

## 2. Materials and Methods

The study was conducted between January 2022 and July 2022 in the city and provinces of Rome, particularly in a few pediatric clinics in the province of Frosinone and in the Pediatric Dentistry Unit of ‘Policlinico Umberto I’ Hospital–‘La Sapienza University’ in Rome, Italy. An anonymous questionnaire was administered to all parents who accompanied their children (aged between 4 and 24 months). The questionnaire validation was verified in a preliminary study [10].

We hypothesized that the samples of children visiting hospitals and clinics could provide a representative local population; therefore, a convenience sample was chosen for this study. 

The study protocol adhered to the Helsinki Declaration guidelines from 1975 and was approved by the Institutional Board of Sapienza University of Rome (Protocol n. 1904-23). The parents of the children were informed about the purpose of the experimental study, and informed consent was obtained before answering the questionnaire.

The draft of the questionnaire was derived from the analysis and study of literature that has contributed over the years to determining the concept and importance of an individual’s health. The final version of the questionnaire included a total of 35 questions related to various topics (Table 1), such as eating habits, oral hygiene, and the usage frequency of homogenized meat, fish, or fruit. 

The inclusion criteria were that the age of the children be between 4 and 24 months old and that the questionnaire be completed for each part.

The utmost attention has been given to the frequency of consumption of meals based on milk, cookies, and sugar. In particular, one of the questions was about parents’ awareness of the real nutritional value of industrial preparations.

Furthermore, another relevant topic addressed in the survey was the choice of the type of water for the newborn and whether it contained a relevant concentration of fluoride, calcium, and other mineral salts.

In addition, parents were asked whether they were influenced by price when buying products for their babies’ alimentation.

The questionnaire was handed over to parents in person, and they were assisted throughout the completion of it but never influenced in their answers. Each patient was associated with a numeric code to treat the data anonymously.

To conclude, a total of 120 questionnaires were collected. After the first screening, 20 questionnaires were excluded because they were incomplete or filled out with conflicting answers. The final 100 questionnaires were analyzed. 

The results were collected using Microsoft^®^ Excel^®^ 2019 MSO database (Version 2403 Build 16.0.17425.20176, USA, 2023); descriptive statistics were developed for each topic using tables and graphs. An analysis of the relationships among the variables has been developed to obtain numerical values that can best represent the distribution of the population in question.

## 3. Results

A total of 120 questionnaires were collected from the pediatric clinics of the province of Frosinone and from the Pediatric Dentistry Unit of ‘Policlinico Umberto I’ Hospital–‘La Sapienza University’ in Rome. After the first screening, 20 questionnaires were excluded because they were incomplete or were filled with conflicting answers. The final 100 questionnaires were analyzed.

Descriptive statistics are presented in Table 1 as frequencies (%) for all the variables represented by the 35 questions asked.

The most represented age ranges in the sample were quite heterogeneous and were 6, 12, and 24 months, accounting for 17%, 11%, and 14%, respectively.

Nearly half of the interviewed parents were working either full time or part time, whereas the remaining 56% did not work at all. Regarding the breastfeeding period, many mothers breastfed for more than a month (57%), but very few of them breastfed for a period of more than 6 months. Question n.5 focuses on the moment in which weaning started; it emerged that mothers of 21 children considered it appropriate to start it between 0 and 4 months, whereas mothers of 78 babies decided to start this process between 5 and 7 months. However, none of the children were weaned after more than 10 months.

The participants of the questionnaire were then asked what type of foodstuff was chosen for their child, and the results showed that the majority of them preferred to feed their newborn with homogenized baby food (47%). As regards to the consumption of homogenized baby foods, the answers to the following question show that 47% of the parents interviewed chose industrial homogenized baby food, while 53% preferred to feed them homemade.

With respect to the eighth question, which concerns the frequency of consumption of meat/fish/rice cream homogenized baby food, the results show that 14 participants consumed them at least 3 times a week, 27 participants consumed them 3 times a week, 15 participants consumed them 6 times a week, and 44 participants consumed them more than 6 times a week.

**Figure 1:**
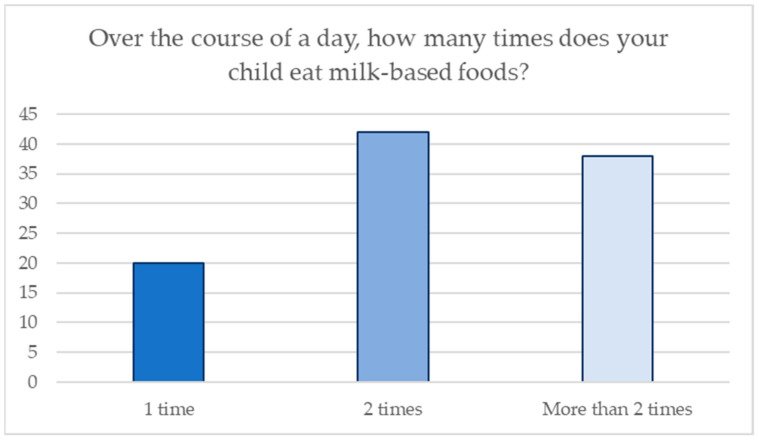
Frequency of eating milk-based foods.

Special attention was given to their milk intake in the analysis. Parents were asked how often they fed their child milk. Figure 1 shows the distribution of the frequency of milk-based food consumption. Furthermore, for question n.11, 68% of the parents added cookies to their children’s milk-based baby food, whereas 32% of them did not.

Parents or caregivers recorded the timing of the eruption of the first tooth. The results show that the first deciduous tooth erupted in 13 children between 3 and 5 months, in 48 of them between 6 and 8 months, in 15 of them between 9 and 12 months, and only in one case it erupted after 12 months, while in the other cases, the first tooth had not yet erupted.

**Figure 2:**
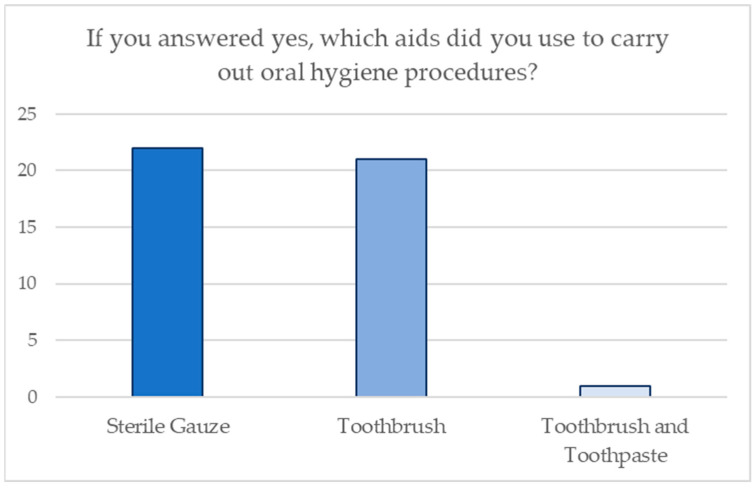
Aids used for domestic oral hygiene procedures.

With respect to maneuvers of oral hygiene, 32 participants claimed to have introduced this practice in their newborn’s first months of life, whereas 68 did not. The parents’ preferred aids for performing oral hygiene procedures are shown in Figure 2.

With respect to fluoride intake, 77% of the interviewees did not use additional fluoride, and among the ones that did use it, 14 of the participants claimed to use it daily, multiple times a day. Coherently, 27% of the interviewees considered it necessary to use additional fluoride, while 73% considered it unnecessary or useless.

Question n. 20 aims to investigate parents’ knowledge about the use of fluoride toothpaste in a specific age group, and the majority considered it appropriate to introduce it after 2 years. Furthermore, 83 “Yes” and 17 “No” were registered to the query of whether a healthy diet can satisfy the need for fluoride in relation to the age of the child.

To further investigate parents’ awareness of fluoride intake, the parents were asked if the amount of fluoride present was a determining factor in choosing one over the others when they purchased water for their family; 37 answered “yes” and 63 answered “no”.

When it comes to their sugar intake, 73% of the parents claimed not to use added sugars when feeding their children, while most of the remaining claimed to use honey (19%).

The parents were also asked whether they paid attention to the labels of those products that were specific for infant nutrition. A total of 74% of the answers were affirmative, whereas the remaining 26% were negative. Parents’ awareness concerning the presence of sugars in meat/fish homogenized baby foods revealed that 34 parents were aware of this issue, whereas 66 people were not. In question n.29, parents were asked if they found it useful to use a pacifier soaked in honey; 10% of them considered it a useful habit, whereas 90% did not. Furthermore, regarding the consumption of sugary drinks as a means of promoting the process of falling asleep, nearly the total disagreed with it (92%).

**Figure 3:**
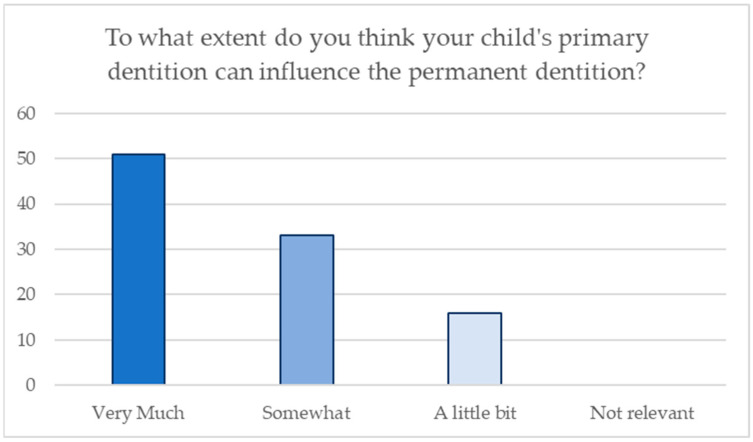
Distribution of answers regarding awareness of the influence of primary dentition on permanent dentition.

The following statistics show the importance of baby teeth compared with permanent teeth according to their parents; Figure 3 shows the distribution of the answers given by the parents.

Furthermore, 93 parents out of 100 were aware of the complications due to the abuse of sugar that their children might face; only 7 of them were unaware of the consequences of this unhealthy habit.

**Figure 4:**
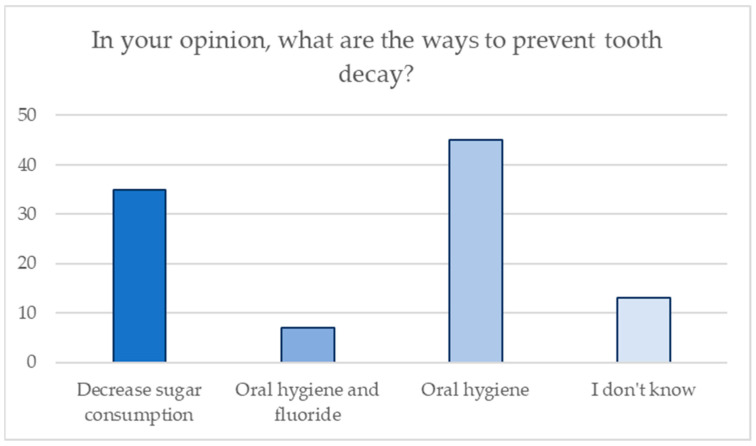
Distribution of answers regarding knowledge of preventive measures for tooth decay.

Similarly, parents were asked whether they had any ideas about how tooth decay could possibly be prevented, and the graph in Figure 4 shows the distribution of the answers given by the parents. 

Question n.34 enlightens parents’ perceptions concerning the effects of prolonged breastfeeding (over 2 years); 32 of them considered it harmful, whereas the remaining 68 did not regard it as an inadequate practice.

Finally, the last question aims to investigate parents’ knowledge about the usefulness of a dental visit before the age of 6, and the great majority agreed that they consider it important.

## 4. Discussion

Considering that carious pathology has a multifactorial etiology (related to eating habits that involve the frequent use of sugars) and that the greatest susceptibility for this pathology extends from early childhood to 6 months, we believe that it was important to evaluate parents’ knowledge and awareness regarding the aforementioned cause–effect relationship that can lead to tooth decay [11,12,13,14,15,16,17]. As expected, the statistical data analysis provides us with worrying results, which, according to the scientific literature, will be some of the major causes of the evolution of carious pathology in the future growth of the pediatric patients taken into consideration.

One of the issues addressed in the questionnaire is breastfeeding, since milk represents the most important and most commonly used nutrient in early childhood. This statement is attributable to the well-known and numerous benefits that milk has, as evidenced in the study “Impact of prolonged breastfeeding on Dental Caries: A population- based birth cohort study”, by Peres K.G. et al. [18]. Research indicates that breastfeeding not only provides numerous systemic health benefits for newborns, such as reducing morbidity and infectious diseases [18], but it also plays a role in dental health by potentially reducing the risk of dental caries [19]. As a matter of fact, evidence suggests that not only does mother’s milk have many nutritional benefits, but breastfeeding also allows the passage of milk from the nipple directly behind the dental arches so as to avoid continuous contact of the teeth with the sugar contained in milk. 

For the aforementioned reason, we have investigated the type, quantity, and quality of milk given to newborns and found out that 43% of the mothers tend to breastfeed the newborn for less than a month or to choose an infant formula instead of the mother’s breast milk. On the one hand, issues such as the inability to breastfeed cannot be discussed since they can be due to the nurse’s health conditions. On the other hand, it is necessary to point out that it is a fairly common practice for perfectly healthy mothers to avoid breastfeeding for logistical or purely aesthetic reasons. 

In the long run, the trend is, therefore, to replace breast milk with artificial milk, which might seem pretty similar to the human one. However, infant formula does not provide the newborn with the essential nutrients that babies need. Moreover, prolonging breastfeeding beyond 2 years is not recommended to prevent complications related to language development. However, 68% of the participants did not consider it a harmful habit.

The World Health Organization has published new guidelines for sugar consumption for adults and children, which recommend limiting the intake of simple sugars to less than 10% of total energy intake [20]. These indications are based on solid scientific evidence, which shows that sugar seems to be associated with an increase in body weight, obesity, and a higher incidence of dental caries. Naturally, these guidelines do not concern sugar found in fresh fruits and vegetables, as well as sugars found in milk, since no adverse effects have been reported from the consumption of these nutrients. Therefore, the study only refers to monosaccharides such as glucose and fructose and disaccharides such as sucrose, which are added to foods and drinks, as well as sugars naturally present in honey, syrups, fruit juice, and industrial baby food products. When it comes to this issue, Chaffee et al. [21] insist on the valid and thoughtful intake control of foods and drinks containing refined sugars, which are proven to be the ones most responsible for the onset of tooth decay, compared to the sugars naturally present in various foods [22]. When it comes to these statements, our analysis shows that 47% of the parents use industrial preparations, and the vast majority of them seem to be unaware of the presence of added sugars in meat and fish baby food despite claiming to carefully read their labels. In addition, some parents even add extra sugary substances such as honey and sucrose to industrial preparations or to homemade ones. On the other hand, results indicate that habits such as the use of a pacifier soaked in honey and the frequent intake of sugary drinks as a useful method of falling asleep are gradually decreasing. Moreover, it frequently happens that the importance of the pediatric patient’s oral hygiene is underrated until the child’s first deciduous teeth start to erupt in the oral cavity or outright until he starts to consume more solid food. Actually, oral hygiene should be practiced from birth in order to prevent bacteria and food residues from producing acidic substances that can cause tooth decay in the long run and release substances that could cause the onset, infection, or inflammation of the gums. Concerning that topic, Wagner et al. [23] insist on the fact that the vast majority of oral diseases, such as tooth decay, defects in the development of dental or periodontal tissues, as well as orthodontic issues, show a complex etiology associated with purely behavioral causes. In these cases, the key to success is to contrast any family habits that tend to be harmful to the patient. In fact, evidence shows that 68% of the parents do not carry out any oral hygiene maneuvers during the baby’s first months of life. In particular, the ones who mostly make use of sterile gauze and a suitable toothbrush, but hardly any of them use cloth or silicone gloves dedicated to the pediatric patient’s fragile oral health.

Although the World Health Organization has published updated guidelines concerning dental health [24], the administration of fluoride to pediatric patients is still a source of controversy. Tooth decay can be naturally prevented and contrasted by the function of saliva, which is improved by the presence of calcium, phosphate, and fluoride. In particular, the lack of fluoride represents a favorable factor for the onset of dental caries; the methods of administration are still uncertain though. The discussion on the aforementioned topic comes from many dentists and pediatricians’ habit of administering systemic fluoride to their patients with the purpose of remineralizing the cavitated regions of the teeth.

Recent studies have evidenced that an excessive dose of fluoride can cause dental fluorosis, a condition that not only can degenerate but that can also involve the skeletal system [25]. In fact, our survey shows that 23% of the parents administer systemic fluoride, which should only be administered in risk categories. Another relevant result that needs to be taken into account is related to the daily recommended fluoride intake in pediatric patients, which is dependent on age. Eighty-three participants claimed that a healthy diet could meet the need for fluoridated substances. On the other hand, 63 parents did not pay attention to the quantity of fluoride present in the water while purchasing it. 

## 5. Conclusions

The comments collected and the statistical data allow us to confirm the initial assumption that inspired this study. After analyzing all the collected data, it is possible to conclude that prevention is the best investment to avoid the onset of carious pathology, which can be possible through parental education, for example, by raising awareness among expectant mothers, through different actions in the territory.

As a matter of fact, not only does parental education have direct implications on the unborn child’s health, but it also has a beneficial effect with respect to nutritional habits, oral hygiene, and more, since the mother can transmit her nutritional education to the child and therefore to the adult of the future. 

Notably, the data for this study were gathered from a specific area, and the sample size was relatively small, which constitutes a limitation of our study, as a hospital-based sample could not serve as a population surrogate. Further research is needed to confirm the abovementioned results.

In conclusion, dental hygienists can fully establish themselves as healthcare figures, capable of implementing educational programs for pregnant women and newborns to promote correct behaviors, improve lifestyles, and protect the general health of individuals.

## Figures and Tables

**Table 1:** Descriptive statistics for dietary and oral health habits and awareness among the N = 100 parents of pediatric patients involved in this study.

Question	N	%
1. How old is your baby (months)?		
4	1	1
5	3	3
6	17	17
7	7	7
8	7	7
9	3	3
10	3	3
11	4	4
12	11	11
13	1	1
14	2	2
15	4	4
16	2	2
17	1	1
18	2	2
19	2	2
20	4	4
21	2	2
22	4	4
23	6	6
24	14	14
2. Do you work outside the home?		
Yes, I’m working full time	23	23
Yes, I’m working part time	21	21
No, I’m not working	56	56
3. Did you breastfeed your baby?		
Yes, I did	57	57
Yes, less than a month	31	31
No, I didn’t	12	12
4. If Yes, how long was the period of breastfeeding (months)?		
1	2	2
2	2	2
3	10	10
4	5	5
5	6	6
6	12	12
7	1	1
8	2	2
9	4	4
10	4	4
11	1	1
12	2	2
13	0	0
14	1	1
15	1	1
16	1	1
17	0	0
18	2	2
19	1	1
5. At what month did weaning start?		
0–4 months	21	21
5–7 months	78	78
8–10 months	1	1
Over the 10 months	0	0
6. What foods do you prefer to feed your baby?		
Ready-to-eat baby food	5	5
Freeze-dried baby food	2	2
Homogenized baby food	47	47
Smoothies	14	14
Other	32	32
7. Which baby foods do you use?		
Commercial homogenized baby food	47	47
Homemade homogenized baby food	57	57
8. In a typical week, how many times does your child eat fruit puree?		
Less than 3 times	11	11
3 times	25	25
6 times	6	6
More than 6 times	48	48
9. In a typical week, how many times does your child eat homogenized meat or fish baby food or rice cream?		
Less than 3 times	14	14
3 times	27	27
6 times	15	15
More than 6 times	44	44
10. Over the course of a day, how many times does your child eat milk-based foods?		
1 time	20	20
2 times	42	42
More than 2 times	38	38
11. Do you add biscuits to milk-based baby food?		
Yes, I do	68	68
No, I don’t	32	32
12. When did the first (milk) tooth erupt?		
3–5 months	13	13
6–8 months	48	48
9–12 months	15	15
Over the 12 months	1	1
It has not yet erupted	1	1
13. Did you perform oral hygiene procedures in the first months of your baby’s life?		
Yes, I did	32	32
No, I didn’t	68	68
14. If you answered Yes, what aids did you use to carry out oral hygiene procedures?		
Sterile gauze	22	22
Toothbrush	21	21
Toothbrush and toothpaste	44	44
15. Did you use gauze and/or cloth or rubber gloves specifically for your child’s home oral hygiene?		
Yes, I did	24	24
No, I didn’t	76	76
16. Does your child receive fluoride supplements?		
Yes, he does	23	23
No, he doesn’t	77	77
17. If you answered Yes, which ones?		
Pediafluor	6	6
Fluormil	4	4
Defluor	0	0
Other	6	6
18. If you answered Yes, how often?		
Once or more times a day	14	14
2 or 3 times a week	5	5
Once a week	2	2
Rarely	2	2
19. Do you think the fluoride supplements are necessary?		
Yes	27	27
No	73	73
20. In your opinion, in what age group should fluoride toothpaste be introduced?		
1 year	19	19
2 years	42	42
3 years	30	30
4 years	2	2
Over 4 years	7	7
21. Do you think that a healthy diet can satisfy the fluoride intake in relation to the age of your child?		
Yes	83	83
No	17	17
22. Does the type of drinking water you usually use at home depend on the amount of fluoride contained in it?		
Yes	37	37
No	63	63
23. Do you use any added sugars in your baby’s diet?		
No, I don’t	73	73
Yes, I use sucrose	2	2
Yes, I use honey	19	19
Yes, I use fructose	4	4
Yes, I use artificial sweeteners	2	2
Yes, I use natural sweeteners	0	0
24. If you answered Yes, how often?		
Frequently	3	3
As needed/if necessary	8	8
Rarely	10	10
25. Does the type of baby foods for your child depend on its price?		
Yes	10	10
No	90	90
26. Do you carefully read the label of baby food products?		
Yes	74	74
No	26	26
27. Did you notice if in homogenized meat baby food or homogenized fish baby food there are also sugars?		
Yes	34	34
No	66	66
28. If your baby cries, do you think he or she is hungry?		
Yes	36	36
No	64	64
29. In your opinion, is the behavior of dipping the pacifier in honey a useful practice?		
Yes	10	10
No	90	90
30. Do you use sugar-sweetened beverages to put your baby to sleep?		
Yes	1	1
Sometimes	7	7
No	92	92
31. To what extent do you think your child’s primary dentition can influence the permanent dentition?		
Very much	51	51
Somewhat	33	33
A little bit	16	16
Not relevant	0	0
32. If your child eats too much sugar, do you know the complications?		
Yes	93	93
No	7	7
33. In your opinion, what are the ways to prevent tooth decay?		
Decrease sugar consumption	35	35
Oral hygiene and fluoride	7	7
Oral hygiene	45	45
I don’t know	13	13
34. Do you think prolonged breastfeeding (over 2 years) is harmful for your child?		
Yes	32	32
No	68	68
35. Do you think a dental visit before the age of 6 is useful for your child?		
Yes	84	84
No	16	16

## Data Availability

The raw data supporting the conclusions of this article will be made available by the authors upon reasonable request.

## References

[1] World Health Organization (2015). European Food and Nutrition Action Plan 2015–2020.

[2] Mensink F., Schwinghammer S.A., Smeets A. (2012). The Healthy School Canteen programme: A promising intervention to make the school food environment healthier. J. Environ. Public Health.

[3] Chen X.X., Xia B., Ge L.H., Yuan J.W. (2016). Beijing da xue xue bao. Yi Xue Ban J. Peking University. Health Sci..

[4] Chong C.Y.L., Bloomfield F.H., O’Sullivan J.M. (2018). Factors Affecting Gastrointestinal Microbiome Development in Neonates. Nutrients.

[5] Venter C., Pereira B., Voigt K., Grundy J., Clayton C.B., Higgins B., Arshad S.H., Dean T. (2009). Factors associated with maternal dietary intake, feeding and weaning practices, and the development of food hypersensitivity in the infant. Pediatr. Allergy Immunol..

[6] Mahoney P. (2015). Dental fast track: Prenatal enamel growth, incisor eruption and weaning in human infants. Am. J. Phys. Anthropol..

[7] Herndon J.B., Tomar S.L., Lossius M.N., Catalanotto F.A. (2010). Preventive oral health care in early childhood: Knowledge, confidence, and practices of pediatricians and family physicians in Florida. J. Pediatr..

[8] Wagner Y., Heinrich-Weltzien R. (2014). Pediatricians’ oral health recommendations for 0- to 3-year-old children: Results of a survey in Thuringia, Germany. BMC Oral Health.

[9] Toumba K.J., Twetman S., Splieth C., Parnell C., van Loveren C., Lygidakis N.A. (2019). Guidelines on the use of fluoride for caries prevention in children: An updated EAPD policy document. Eur. Arch. Paediatr. Dent..

[10] Straccamore L. (2019). Experimental Study on the Correlation between Developmental Nutrition and Oral Health. Master’s Thesis.

[11] Cogulu D., Saglam C. (2022). Genetic aspects of dental caries. Front. Dent. Med..

[12] Petersen P.E., Bourgeois D., Ogawa H., Estupinan-Day S., Ndiaye C. (2005). The global burden of oral diseases and risks to oral health. Bull. World Health Organ..

[13] Renuka P., Pushpanjali K., Sangeetha R. (2013). Review on “Influence of host genes on dental caries”. IOSR J. Dent. Med. Sci..

[14] Slayton R.L., Cooper M.E., Marazita M. (2005). Tuftelin, mutans streptococci, and dental caries susceptibility. J. Dent. Res..

[15] Harris N.O., Garcia-Godoy F. (2004). Primary Preventive Dentistry.

[16] Polimeni A. (2019). Odontoiatria Pediatrica.

[17] Anil S., Anand P.S. (2017). Early Childhood Caries: Prevalence, Risk Factors, and Prevention. Front. Pediatr..

[18] Peres K.G., Nascimento G.G., Peres M.A., Mittinty M.N., Demarco F.F., Santos I.S., Matijasevich A., Barros A.J.D. (2017). Impact of Prolonged Breastfeeding on Dental Caries: A Population-Based Birth Cohort Study. Pediatrics.

[19] Tham R., Bowatte G., Dharmage S., Tan D., Lau M., Dai X., Allen K., Lodge C. (2015). Breastfeeding and the risk of dental caries: A systematic review and meta-analysis. Acta Paediatr..

[20] World Health Organization (2015). Guideline: Sugars Intake for Adults and Children.

[21] Chaffee B.W., Feldens C.A., Rodrigues P.H., Vitolo M.R. (2015). Feeding practices in infancy associated with caries incidence in early childhood. Community Dent. Oral Epidemiol..

[22] Feldens C.A., Rodrigues P.H., De Anastacio G., Vitolo M.R., Chaffee B.W. (2018). Feeding frequency in infancy and dental caries in childhood: A prospective cohort study. Int. Dent. J..

[23] Wagner Y., Heinrich-Weltzien R. (2017). Risk factors for dental problems: Recommendations for oral health in infancy. Early Hum. Dev..

[24] World Health Organization (2023). Global Oral Health Status Report: Towards Universal Health Coverage for Oral Health by 2030. Regional Summary of the African Region.

[25] Everett E.T. (2011). Fluoride’s effects on the formation of teeth and bones, and the influence of genetics. J. Dent. Res..

